# Estimating loss in quality of life associated with asthma-related crisis events (ESQUARE): a cohort, observational study

**DOI:** 10.1186/s12955-019-1138-5

**Published:** 2019-04-11

**Authors:** Christina-Jane Crossman-Barnes, Tracey Sach, Andrew Wilson, Garry Barton

**Affiliations:** Faculty of Medicine and Health Sciences, Norwich Medical School, University of East Anglia, Norwich, NR4 7TJ UK

**Keywords:** Asthma, Crisis event, Quality of life, Utility estimations, UK

## Abstract

**Background:**

Evidence of quality of life implications of asthma attacks are limited, particularly when measured on a utility scale, which enables calculating Quality-Adjusted Life-Years (QALYs) and comparisons with other health conditions and services. Therefore, this study sought to estimate the utility loss associated with an asthma-related crisis event (accident and emergency (A&E) attendance or hospital admission).

**Methods:**

Participants were recruited in a cohort study from A&E and hospital admissions at three UK hospitals. They completed the EuroQol-5 Dimensions 5-Level (EQ-5D-5 L), Asthma Quality of Life Questionnaire (AQLQ), Time trade-off (TTO), and peak flow and symptom diary over 8 weeks, where three different methods (EQ-5D-5 L, AQLQ, and TTO), were used to estimate utilities. The mean difference between two time points were estimated using the Wilcoxon signed rank test.

**Results:**

From baseline to week 8, mean increases (95% CI) were estimated to be 0.086 (0.019–0.153), 0.154 (0.112–0.196) and 0.132 (0.063–0.201) for EQ-5D-5 L, AQL-5D (preference-based measure derived from AQLQ), and TTO respectively over 8 weeks (*p* < 0.01).

**Conclusion:**

Asthma crisis events are estimated to be associated with a mean utility loss of between 0.086 and 0.132. The utility decrement can be used to assign values to asthma-related crisis events, which can enhance economic evaluations.

**Trial registration:**

NCT02771678. Registered 13 May 2016.

**Electronic supplementary material:**

The online version of this article (10.1186/s12955-019-1138-5) contains supplementary material, which is available to authorized users.

## Introduction

Asthma has a prevalence of over 300 million people worldwide, and it can be a severe and life threatening condition [1]. Each week in England and Wales, there are 1400 asthma patients hospitalized, and direct costs are estimated to amount to over £1 billion [2, 3]. The onset of asthma symptoms can develop gradually or suddenly lead to an attack, leading to impaired quality of life [4].

Generic and disease-specific patient reported outcome measures (PROMs) can be used to measure an individual’s health state. Many asthma-related studies have used PROMs [4–6], however, they mostly measure PROMs at specific time points (e.g. baseline and 6 months) and (as they have no information as to what happens in between) assume linear interpolation (a gradual straight line change) between such points [7]. Others have argued, however, that such methods may not capture the loss in quality of life associated with particular health events [8–10]. Asthma attacks are unpredictable, and if they occur between specific time points of measurement (e.g. baseline and 6 months), the loss associated with an event may not be captured and consequently, overall quality of life could potentially be overestimated. Conversely, if a follow up time point occurred during an asthma-related event, then using the linear interpolation method could result in underestimating overall quality of life. Therefore, with the above method, there is a potential for the utility estimates (a scale on which 0 is equivalent to death and 1 is full health), QALY values, and cost-effectiveness to be inaccurate, with the possibility that treatments could be recommended for provision when they are not in fact cost-effective, or not recommended when they are in fact cost-effective.

In light of the above, the objective of this study is to estimate the loss in health-related quality of life and enable QALY values to be estimated via an alternative method associated with an asthma crisis event (A&E attendance or hospital admission). This will enable studies that capture outcomes in terms of A&E attendances or hospital admissions to also convert their outcomes, akin to mapping [11, 12], into QALY estimates. This is in line with NICE methods recommendations [13, 14], and it will enable recommendations about whether NHS provision of particular interventions constitutes value for money to be more readily made.

## Methods

### Study design

The ESQUARE study was an 8 week prospective, observational cohort study. The target sample size was between 100 and 200 participants, with consideration of the retention rate [15] and previous literature [16–18]. Originally the aim was to recruit 100 participants informed by the literature. However, due to the large unforeseen number of participants who did not complete the study due to withdrawals or loss to follow up, the sample size was increased to 200 participants to aid with retaining participants who would complete the whole study.

### Participant recruitment

Participants were screened and recruited in the UK from the Norfolk and Norwich University Hospital in Norwich, the Queen Elizabeth Hospital in Birmingham and the Aberdeen Royal Infirmary in Aberdeen. Approval was granted by the Cambridge South Research Ethics Committee and recruitment took place between May 2016 and May 2017. Participants were eligible if they had an asthma-related crisis event and a diagnosis of asthma alone, or asthma with Chronic Obstructive Pulmonary Disease (COPD) or asthma with a respiratory infection. They also had to be ≥18 years old, able to speak English and give informed consent. Participants were excluded if they had life threatening hypoxaemia (unable to speak in short sentences), were unable to complete questionnaires unaided, or if they had already participated in the study.

### Study procedures

The investigators and respiratory staff identified and consented participants from the daily hospital triage. After obtaining consent, participants were asked to complete baseline questionnaires in the presence of a researcher (demographics questionnaire, EQ-5D-5 L [19], AQLQ [20], and TTO [21]), and given two packs of questionnaires to complete for the 8 weeks. The first pack included the EQ-5D-5 L, AQLQ and a peak flow and symptom diary (see below), to be completed for the first four weeks. The second pack (posted at week 3 of the study), contained the same aforementioned questionnaires, to be completed for the last four weeks. Throughout the 8 week period the EQ-5D-5 L was requested to be completed weekly, the AQLQ every 4 weeks, the TTO every 4 weeks and the peak flow and symptom diary daily. Participants were also telephoned or seen face to face (depending on locations and schedules of routine hospital follow-ups) at weeks 3, 4, 6, and 8 to monitor progress and asked further questions regarding adverse events, changes in asthma medications, comorbidities, smoking status and to complete the TTO. Participants returned the completed questionnaires in freepost envelopes provided, and received £30 in vouchers to thank them for their time participating in the study. (See Additional file 1).

### Outcome measures

Table 1 shows the array of questionnaires and forms requested at different time points of the study.

**Table 1:** Time and events during the study

Questionnaires/Forms	Baseline	Week 1	Week 2	Week 3	Week 4	Week 5	Week 6	Week 7	Week 8
Researcher with participant completion									
Consent form	X								
Patient and GP details form	X								
Time Trade Off	X				X				X
Participant completion									
Demographics questionnaire	X								
EuroQol-5 Dimensions-5 Level Questionnaire	X	X	X	X	X	X	X	X	X
Asthma Quality of Life Questionnaire	X				X				X
Peak flow and symptoms diary	Completion of this diary was requested every day from baseline through to week 8								

#### EuroQol-5 dimensions 5 levels (EQ-5D-5 L)

The EQ-5D is a widely used questionnaire and is recommended by NICE for use in economic evaluation studies [22], and the EQ-5D-5 L is currently undergoing research for its suitability in technology appraisals [23]. The EQ-5D-5 L is a generic questionnaire composed of 5 dimensions (mobility, self-care, usual activities, pain/discomfort, and anxiety/depression). Each dimension has 5 levels (no problems, slight problems, moderate problems, severe problems, extreme problems/unable), and describes the participants’ health on the day the questionnaire is completed [24]. If all 5 questions are answered, the responses are converted into a health index score to generate a utility value on a scale of 0 (death) to 1 (full health) [25]. In addition, there is also a Likert scale called the Visual Analogue Scale (VAS), ranging from 0 (the worst health you can imagine) to 100 (the best health you can imagine), where the participant records a value which best describes their health.

#### Asthma quality of life questionnaire (AQLQ)

The AQLQ is a disease-specific questionnaire consisting of 32 asthma-related questions, based on the participants’ last 2 weeks. There are 7 different response choices for each question ranging from 1 (e.g. all of the time) to 7 (e.g. none of the time) [20]. Additionally, responses to five questions around sleep, concern, breath, pollution and activity were used from the AQLQ to develop the preference based measure called the AQL-5D. Associated utility values between 0 and 1 were derived based on using the TTO from a random sample of the UK population [26]. It is recommended in the literature that disease-specific instruments should be used in conjunction with generic HRQL instruments [22]. Therefore, both the EQ-5D-5 L and AQL-5D were used together to inform this study, with the added benefit of both questionnaires having five dimensional levels.

#### Time trade off (TTO)

In line with previous work, the TTO method used was modified slightly [27]. The two options are typically *the condition of interest* and *full health*. In this case, the two options were *current asthma health state* and *well controlled asthma*. The latter option was chosen in order to enable one to specifically estimate the loss in quality of life associated with an asthma-related crisis event, without having to adjust for any co-morbidities that may be present. The TTO had an advantage over using the quality of life questionnaires (e.g. EQ-5D-5 L), because it was designed as such with a view to be able to identify whether the participant has returned back to their well-controlled asthma state, therefore reducing the possibility of underestimating the loss in quality of life associated with an asthma crisis event if they have still not returned to a well-controlled state by week 8.

The chosen life expectancy for the TTO was that of the general population [28], taking account of the individuals’ age and sex, as if asthma is well controlled then the individual should be able to live a normal life, equivalent to a healthy person (dependent of other comorbidities) [29].

The TTO was asked at baseline by a face to face consultation, initially using a visual on a laptop to aid the explanation of the TTO. The TTO follow-ups conducted at week 4 and week 8 of the study were provided either face to face at the participant’s routine hospital appointment or over the telephone. The initial face to face consultation at baseline was intended to help the participants remember the visual displayed for their telephone consultation. For this study, the iterative questioning of the TTO began at the mid-point of the participant’s estimated remaining average life expectancy, with incremental movements during the TTO process by 10% of their estimated remaining average life expectancy rounded to the nearest 10.

#### Peak expiratory flow (PEF) and symptoms diary

A diary was used to record participants’ PEF (recorded morning and evening) and symptom severity in relation to three questions from the Royal College of Physicians (RCP) [30]. The three questions were as follows, where the response options were no symptoms, slight, moderate or severe symptoms:

1. Have you had difficulty sleeping because of your asthma?
2. Have you had your usual asthma symptoms during the day (cough, wheeze, breathlessness, chest tightness)?
3. Has your asthma interfered with your usual activities (e.g. housework, childcare, work, school etc.)?

### Statistical analysis

Double data entry was conducted for 10% of the data collected, where there was only 5 out of 1600 (0.31%) data points that differed between both data sets, indicating that accuracy was high. Baseline and descriptive statistics were explored from available cases using STATA (v.12) and Microsoft Excel (2016) software packages. Mean changes and ranges of the utility values and scores for each follow up time point were computed. As the data followed a non-normal distribution, the Wilcoxon signed-rank test at the 5% statistical level was conducted for available case analysis. The QALY loss was also estimated, for each of the EQ-5D-5 L, AQLQ-5D and TTO, by taking account of the utility values and the time points at which these questionnaires were asked. The predicted PEF was estimated using the mini-wright online PEF calculator [31]. Response rates, floor and ceiling effects, were also tabulated at different time points of the study.

## Results

### Demographics

**Fig. 1:**
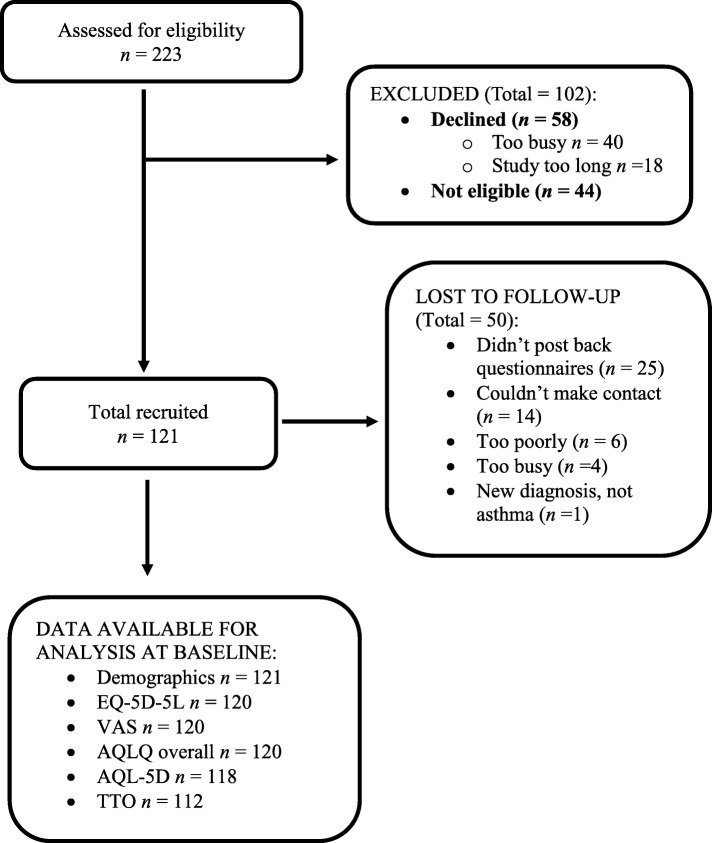
Recruitment flow diagram

Across all three hospital sites, 223 participants were screened for eligibility. Of these, 121 were recruited into the study, with 42 (35%) lost to follow up (8 week scores not completed) and 8 (7%) withdrawals (Fig. 1).

The mean age of participants was 50 years old, with 26.5% male and 95.8% white (Table 2).

**Table 2:** Baseline characteristics

Demographics	*N* = 121
Age (mean, years)	49.68
Height (mean, cm)	167.22
Weight (mean, kg)	85.54
Gender (%)	
Male	26.45
Female	73.55
Ethnicity (%)	
White	95.83
Mixed White and Black	0.83
White Other	3.33
Smoking Status (%)	
Never	42.50
Non-Smoker	1.67
Smoker	15.00
Ex-Smoker	40.83
Highest Level of Education (%)	
School	47.06
College	33.61
Degree	19.33
Employment status (%)	
Full-time	27.50
Part-time	15.83
Retired	28.33
Stay at home parents	7.50
Student	3.33
Unemployed	17.50

At baseline, self-reported data from the demographics questionnaire indicated that the most frequent route of entry into hospital was by GP or nurse referral (42%), or by ambulance (42%), and for 60% of participants, the peak of their asthma event occurred before hospital. Medical records reported that the average length of stay was 5.0 days. The response rates for the EQ-5D-5 L, TTO, AQLQ symptoms, emotional and environmental scores showed evidence of ceiling effects **(**Table 3). The TTO had the highest percentage of 18.8% for ceiling effects, as 21 participants reported that they were not willing to reduce their life expectancy in exchange for an improvement in asthma control. The baseline response rates ranged from 97.5 to 100.0%.

**Table 3:** Baseline statistics for each quality of life questionnaire

Item	N	Mean	SD	Range	Response rates	Floor effects	Ceiling effects
EQ-5D-5 L (utility)	120	0.635	0.274	−0.102 to 1.00	99.2%	0.00%	8.30%
VAS score	120	45.7	19.3	5.00 to 90.0	99.2%	0.00%	0.00%
AQLQ overall score	120	3.28	0.963	1.18 to 5.30	99.2%	0.00%	0.00%
AQLQ Symptoms score	121	2.81	1.06	0.00 to 5.50	100.0%	0.00%	2.50%
AQLQ Activity score	121	3.51	1.05	0.00 to 5.82	100.0%	0.00%	0.00%
AQLQ Emotional score	121	3.14	1.51	0.00 to 7.00	100.0%	0.00%	4.10%
AQLQ Environmental score	121	4.04	1.52	0.00 to 7.00	100.0%	0.00%	1.70%
AQL-5D (utility)	118	0.608	0.128	0.450 to 0.935	97.5%	0.00%	0.00%
TTO (utility)	112	0.626	0.277	0.100 to 1.00	100.0%^a^	0.00%	18.8%

^a^The response rate is based on the denominator being 112 due to only the participants based at the Norfolk and Norwich University Hospital (NNUH) being asked the TTO questions. All of the other response rates for the PROMS were based on the denominator being 121 as this was the total number recruited across all hospital sites where each participant was asked to complete PROM questionnaires. Ranges for PROMs: EQ-5D-5 L (−0.281 to 1); EQ-5D VAS (0 to 100); AQLQ (0 to 7); AQL-5D (0 to 1); TTO (0 to 1)

### Utility and QALY loss

Table 4 shows the utility and score results at all the time points. The preference based questionnaires (EQ-5D-5 L, AQL-5D, and TTO) showed statistical significant differences (*p* < 0.01) between baseline and week 8 (Table 5). Likewise, the PROMs observed between baseline and week 4, were also all statistically significant (*p* < 0.01). (Table 6) However, for the PROM scores where the comparison was between week 4 and week 8, only the AQLQ overall score and AQL-5D were statistically significantly different (*p* < 0.05) (see Additional file 2).

**Table 4:** Mean utility values and scores at weekly time points shown between baseline and week 8

	Baseline Mean (CI)	Week 1 Mean (CI)	Week 2 Mean (CI)	Week 3 Mean (CI)	Week 4 Mean (CI)	Week 5 Mean (CI)	Week 6 Mean (CI)	Week 7 Mean (CI)	Week 8 Mean (CI)	*p* Value
EQ-5D-5L	*N* = 120	*N* = 81	*N* = 75	*N* = 74	*N* = 71	*N* = 65	*N* = 65	*N* = 64	*N* = 64	*N* = 64
	0.64 (0.59,0.68)	0.65 (0.59,0.70)	0.70 (0.65,0.75)	0.72 (0.66,0.77)	0.74 (0.68,0.80)	0.76 (0.71,0.82)	0.77 (0.71,0.83)	0.78 (0.73,0.84)	0.72 (0.65,0.80)	*P*<0.007**
VAS	*N* = 120	*N* = 81	*N* = 75	*N* = 75	*N* = 73	*N* = 66	*N* = 65	*N* = 64	*N* = 64	*N* = 64
	45.68 (42.34,49.02)	57.70 (53.36,62.05)	60.79 (56.03,65.54)	63.21 (59.01,67.42)	65.95 (61.01,70.88)	68.09 (63.36,72.83)	68.75 (64.19,73.32)	71.56 (66.99,76.14)	67.88 (62.58,73.17)	*P*<0.000**
AQLQ overall	*N* = 120				*N* = 70				*N* = 65	*N* = 65
	3.28 (3.11,3.45)				4.09 (3.76,4.42)				4.48 (4.13,4.83)	*P*<0.000**
AQLQ Symptoms	*N* = 121				*N* = 85				*N* = 66	*N* = 66
	2.81 (2.62,3.00)				3.33 (2.89,3.77)				3.64 (3.10,4.18)	*P*<0.003**
AQLQ Activity	*N* = 121				*N* = 85				*N* = 66	*N* = 66
	3.51 (3.33,3.69)				3.32 (2.90,3.73)				3.68 (3.17,4.19)	*P* <0.044*
AQLQ Emotional	*N* = 121				*N* = 85				*N* = 66	*N* = 66
	3.14 (2.87,3.40)				3.36 (2.88,3.84)				3.72 (3.14,4.31)	*P* <0.041*
AQLQ Environmental	*N* = 121				*N* = 85				*N* = 66	*N* = 66
	4.04 (3.76,4.31)				3.63 (3.12,4.13)				3.91 (3.34,4.47)	*P* <0.089
AQL-5D	*N* = 118				*N* = 70				*N* = 64	*N* = 62
	0.61 (0.59,0.63)				0.69 (0.65,0.73)				0.74 (0.69,0.78)	*P*<0.000**
TTO	*N* = 112				*N* = 87				*N* = 80	*N* = 80
	0.63 (0.58,0.68)				0.82 (0.76,0.88)				0.79 (0.72,0.85)	*P*<0.000**

Wilcoxon signed-rank test shown for the mean change between baseline and week 8

Bootstrap (1000 replications) confidence intervals displayed at each time point

**p-value < 0.01 therefore statistically significant at the 1% level

*p-value < 0.05 therefore statistically significant at the 5% level

**Table 5:** Mean changes in utility and score values between baseline and week 8 (available case analysis)

Outcome measure	N	Baseline Mean ± SD	8 weeks Mean ± SD	Mean difference (95% CI)	*P*-value
EQ-5D-5 L (utility)	64	0.639 ± 0.267	0.725 ± 0.294	0.086 (0.153 to 0.019)	0.007**
VAS (score)	64	48.81 ± 18.58	67.88 ± 22.03	19.06 (25.69 to 12.44)	0.000**
AQLQ overall (score)	65	3.20 ± 0.955	4.48 ± 1.50	1.28 (1.60 to 0.963)	0.000**
AQL-5D (utility)	62	0.582 ± 0.120	0.736 ± 0.178	0.154 (0.196 to 0.112)	0.000**
TTO (utility)	80	0.655 ± 0.273	0.787 ± 0.295	0.132 (0.201 to 0.063)	0.000**

Wilcoxon signed-rank test

**p-value < 0.01 therefore statistically significant at the 1% level

**Table 6:** Mean change in utility and score values between baseline and week 4 (available case analysis)

Outcome measure	N	Baseline Mean ± SD	4 weeks Mean ± SD	Mean difference (95% CI)	*P*-value
EQ-5D-5 L (utility)	71	0.613 ± 0.275	0.740 ± 0.264	0.127 (0.193 to 0.061)	0.000**
VAS (score)	73	47.38 ± 20.08	65.95 ± 21.42	18.56 (23.40 to 13.72)	0.000**
AQLQ (score)	70	3.16 ± 0.980	4.09 ± 1.48	0.929 (1.19 to 0.666)	0.000**
AQL-5D (utility)	69	0.589 ± 0.126	0.687 ± 0.174	0.099 (0.134 to 0.063)	0.000**
TTO (utility)	87	0.650 ± 0.278	0.820 ± 0.264	0.170 (0.243 to 0.097)	0.000**

Wilcoxon signed-rank test

**p-value < 0.01 therefore statistically significant at the 1% level

The estimated QALY loss over the first eight weeks for an asthma-related crisis event for the EQ-5D-5 L, AQL-5D and TTO is 0.007, 0.012 and 0.010 QALYs respectively, with the assumption of linear interpolation between baseline and week 8 for the available case analysis.

### Peak flow and asthma symptoms

The mean difference between week 8 and baseline was 64 L/min, which was a statistically significant difference (*p* < 0.05). The mean best and predicted PEF were 377 and 490 respectively, indicating that participants PEF had not returned to their best or predicted values by week 8 (see Additional file 3).

On average, all participants who responded to the RCP symptom question (*N* = 60) reported mild symptoms approximately one week after consent into the study (see Additional file 4). However, 6.6% of responding participants reported another asthma-related hospitalization during the study and 28.9% reported changes to their medications. New comorbidities and smoking status arose within the 8 weeks for some participants, averaging at 2.5 and 3.3% respectively.

## Discussion

This study explored quality of life in people with acute asthma who attended A&E or were admitted to hospital with an asthma attack. The aim was to estimate the associated utility/QALY loss for the aforementioned patient population, to enable studies to use these estimations, and aid their decision about the best value for money for particular interventions. The mean differences between week 8 and baseline were 0.086, 0.154 and 0.132 for the EQ-5D-5 L, AQL-5D and TTO utilities respectively. Assuming linear interpolation, their corresponding QALY loss estimates over the first 8 weeks were 0.007, 0.012 and 0.010 QALYs respectively.

There are a number of strengths from this study. The participants were recruited during one year from three hospital sites, which enhanced the generalisability of the collected data for the asthma population. Several PROMs were used in this study to gain a more comprehensive perspective on quality of life in people with acute asthma. A limitation is however, that the peak of the asthma-related crisis event could have occurred before A&E attendance or hospital admission, meaning that the baseline score may not equate to the worst point and the reported change scores therefore represent an underestimate of the loss in quality of life associated with an asthma crisis event. Secondly, since the participants had not yet returned to their normal PEF at week 8 of the study, the estimation in quality of life associated with an asthma crisis event could have also been further underestimated. Thirdly, participants were excluded if they were unable to complete the questionnaires unaided, which could have potentially excluded the extremely severe asthma participants, again meaning that the estimated loss in utility could have been underestimated. Fourthly, the retention rate was problematic and the sample size was small, with a large proportion of participants lost to follow up, despite phone call reminders. The low retention rate could have been due to the study length being too long [32], but may also be related to the population in question e.g. asthmatics have been shown to often be non-compliant with attending clinic appointments [33]. Finally, we should acknowledge that though there is an argument for correcting TTO scores for time preference [34], we have not done so as this is not generally undertaken and there is no one agreed correction factor [35].

An earlier four week study used the EQ-5D-3 L in asthmatics, and estimated the mean utility loss associated with an asthma hospitalization as 0.20 [32], which differs from our mean findings of 0.13. However, the earlier study used the EQ-5D-3 L, [32], compared to this study using the EQ-5D-5 L, and though the 5 level aims to improve sensitivity [24] it has been shown that the 3 L version can lead to higher utility gains compared to the EQ-5D-5 L [36]. Some of the difference observed could have also occurred, as in the previous study patients were not experiencing an asthma attack at the point at which they were recruited into the study [32], as recruitment was from outpatient clinics and primary care. Accordingly, their score at the time of event was compared to their pre-admission score, whereas our comparison was the 8 week score. It may be that in our study they had not recovered to their pre-admission score by that point, meaning that our aforementioned estimates may represent conservative estimates, as to the loss in quality of life associated with an asthma crisis event.

The ceiling effects for the TTO (anchor: well-controlled asthma) were 18.8% at baseline, and increased to 51.7 and 51.3% at weeks 4 and 8 respectively (see Additional files 5 and 6). Here participants report not being willing to trade reductions in life expectancy in exchange for improvements in asthma control. Previously, it has been suggested that willingness to trade maybe associated with participant characteristics such as marital status, age and family circumstances [37, 38]. Here improvements (which largely occurred by week 4) may also explain why participants were less likely to trade any life years at weeks 4 and 8.

A particular application of this work is that the estimated QALY loss can be used to enable other studies [7, 39], which have estimated outcomes in terms of asthma-related hospital admissions/crisis events, to convert their results into a QALY score. With associated cost data, this will enable the cost per QALY gain to be estimated, and after taking account of guidance about value for money [40], recommendations about provision can be made, where this would not have otherwise been possible. Also, as outlined in the introduction, researchers and policy makers should be mindful that the above described method, which is akin to mapping [12], has the potential to produce different results to those based on QALY values derived from follow-up at fixed time points with linear interpolation. Comparison of these two methods therefore represents a potential avenue for further research.

## Conclusion

To conclude, this study estimated the utility loss associated with asthma-related crisis events, where most of the loss was observed within the first four weeks. The EQ-5D-5 L, AQL-5D and TTO showed mean utility changes between baseline and week 8 of 0.086, 0.154 and 0.132 respectively (*p* < 0.01). In turn, these values can facilitate the estimation of cost-effectiveness, where estimates of the associated QALY loss are combined with data on the number of asthma-related crisis events.

## Additional files

Additional file 1: Flow chart of study design. (PDF 248 kb)

Additional file 2: Mean changes in utility and score values between week 4 and week 8 (available case analysis). (PDF 151 kb)

Additional file 3: Mean peak expiratory flow at daily time points. (PDF 164 kb)

Additional file 4: Mean scores for difficulties sleeping, symptoms and activities at daily points. (PDF 165 kb)

Additional file 5: Week 4 statistics for each quality of life questionnaire. (PDF 151 kb)

Additional file 6: Week 8 statistics for each quality of life questionnaire. (PDF 151 kb)

## Abbreviations

A&E: Accident and emergency
AQL-5D: Asthma Quality of Life Utility Index-5 Dimensions
AQLQ: Asthma Quality of Life Questionnaire
CLAHRC: Collaboration for Leadership in Applied Health Research & Care
EQ-5D-3 L: EuroQol-5 Dimensions – 3Level
EQ-5D-5 L: EuroQol-5 Dimensions-5 Level
GP: General Practitioner
NHS: National Health Service
NICE: The National Institute for Health and Care Excellence
NIHR: National Institute for Health Research
PEF: Peak Expiatory Flow
PROMs: Patient reported outcome measures
QALY: Quality Adjusted Life Year
RCP: Royal College of Physicians
TTO: Time trade-off
UK: United Kingdom
VAS: Visual analogue scale
