# Supplementary figures and images for: Estimating loss in quality of life associated with asthma-related crisis events (ESQUARE): a cohort, observational study
Source: Health Qual Life Outcomes. 2019 Apr 11;17:58. doi: 10.1186/s12955-019-1138-5 (PMC6458613; doi:10.1186/s12955-019-1138-5)

Figure 1: Flow chart of study design

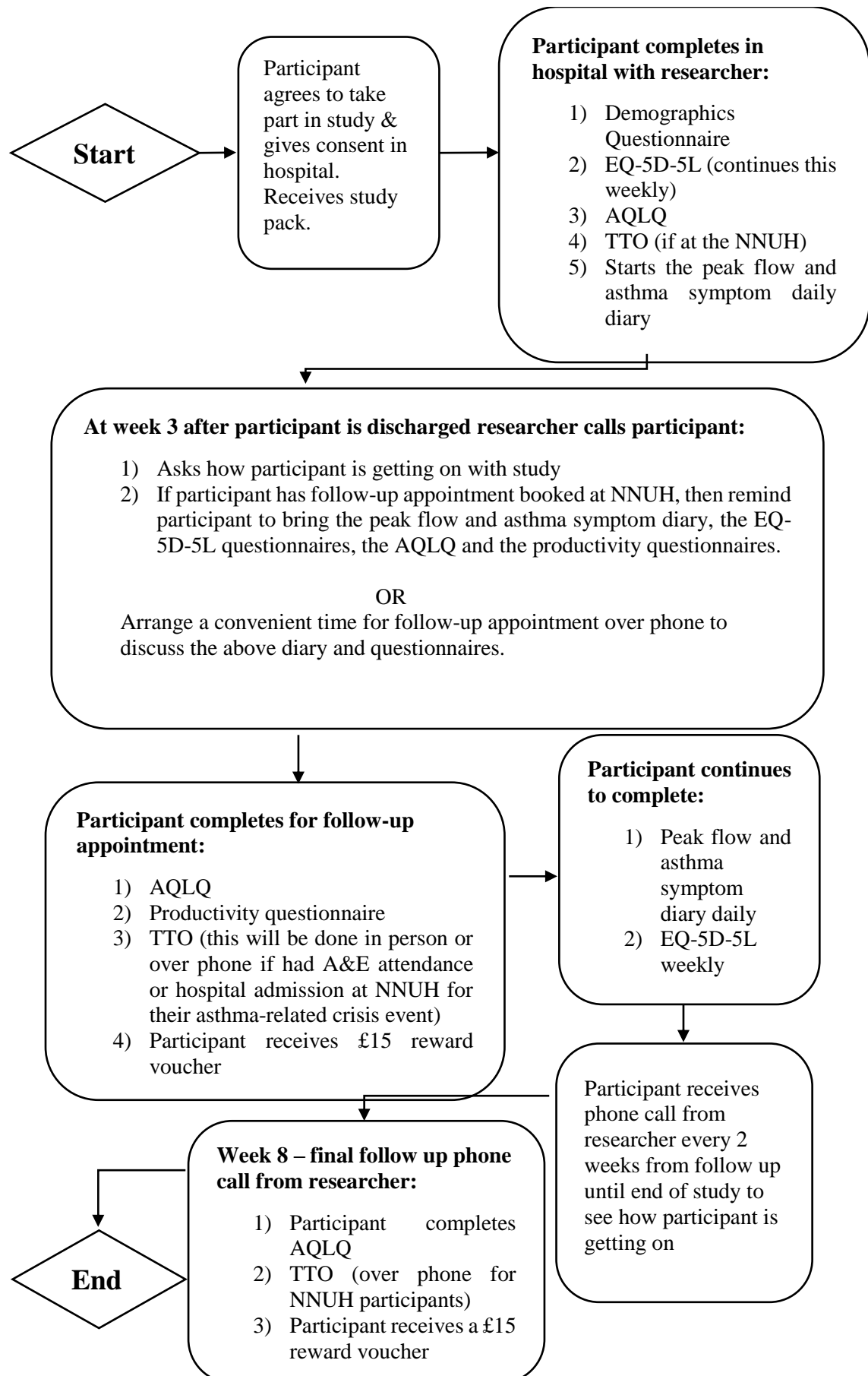

Supplement: Supplementary file 1 — Flow chart of study design. (PDF 248 kb) [file 12955_2019_1138_MOESM1_ESM.pdf]

*Figure 1: Mean Peak Expiratory Flow at daily time points*

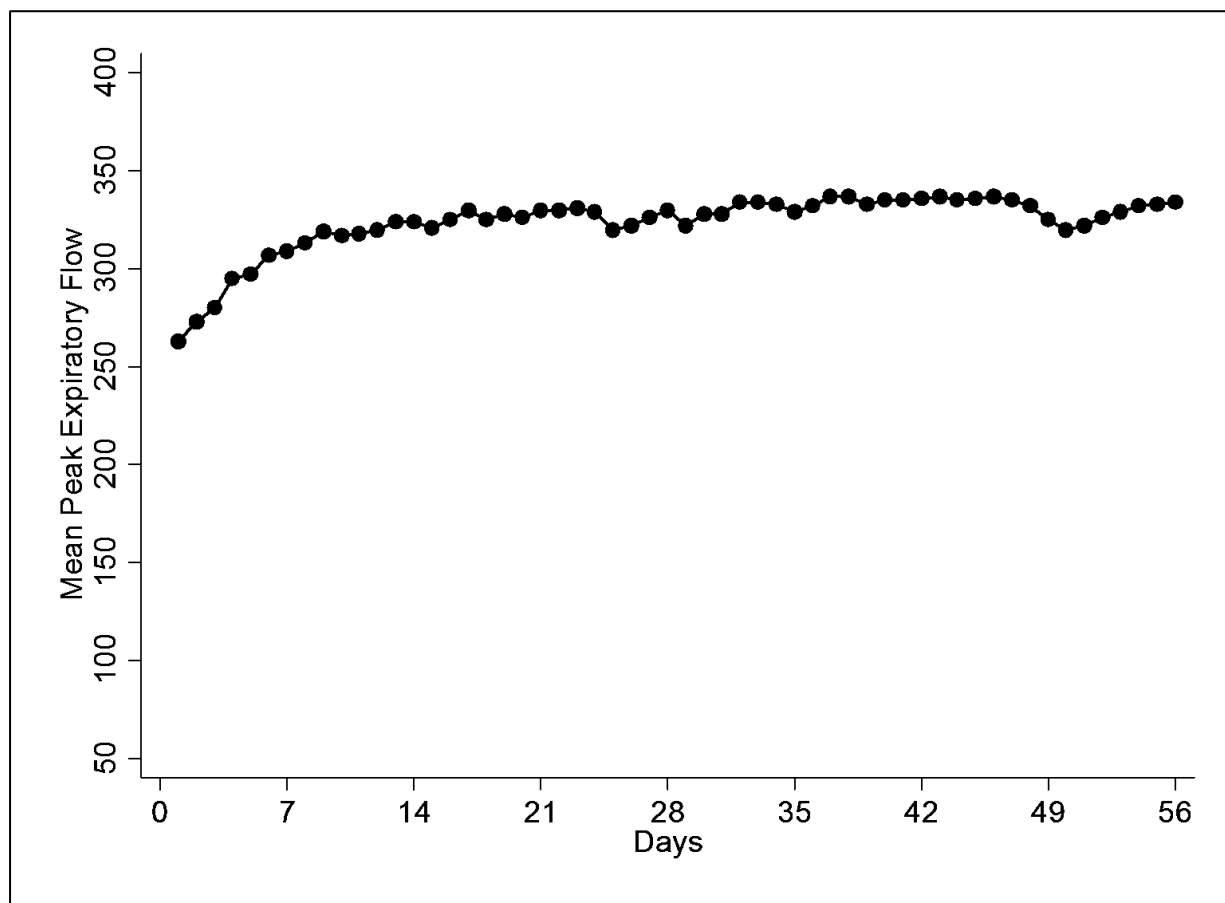

Supplement: Supplementary file 3 — Mean peak expiratory flow at daily time points. (PDF 164 kb) [file 12955_2019_1138_MOESM3_ESM.pdf]
