# Supplementary material for: Estimating loss in quality of life associated with asthma-related crisis events (ESQUARE): a cohort, observational study
Source: Health Qual Life Outcomes. 2019 Apr 11;17:58. doi: 10.1186/s12955-019-1138-5 (PMC6458613; doi:10.1186/s12955-019-1138-5)
Supplement: Supplementary file 2 — Mean changes in utility and score values between week 4 and week 8 (available case analysis). (PDF 151 kb) [file 12955_2019_1138_MOESM2_ESM.pdf]

Table 1: Mean changes in utility and score values between week 4 and week 8 (available case analysis)

**Outcome measure**	**N**	**4 weeks** ***Mean ± (SD)***	**8 weeks** ***Mean ± (SD)***	**Mean difference (95% CI)**	**P-value**
**EQ-5D-5L (utility)**	59	0.745 ± 0.255	0.720 ± 0.302	0.025 (0.033 to 0.082)	0.394
**VAS (score)**	61	67.41 ± 20.31	68.51 ± 22.13	1.10 (4.60 to 2.41)	0.533
**AQLQ (score)**	57	4.23 ± 1.52	4.52 ± 1.55	0.291 (0.536 to 0.046)	0.021*
**AQL-5D (utility)**	56	0.700 ± 0.179	0.740 ± 0.181	0.040 (0.078 to 0.002)	0.041*
**TTO (utility)**	76	0.813 ± 0.268	0.794 ± 0.290	-0.019 (-0.047 to 0.086)	0.561

Wilcoxon signed-rank test
*p-value <0.05 therefore statistically significant at the 5% level

Estimating loss in quality of life associated with asthma-related crisis events (ESQUARE): a cohort, observational study
Additional file 2
