# Supplementary material for: Estimating loss in quality of life associated with asthma-related crisis events (ESQUARE): a cohort, observational study
Source: Health Qual Life Outcomes. 2019 Apr 11;17:58. doi: 10.1186/s12955-019-1138-5 (PMC6458613; doi:10.1186/s12955-019-1138-5)
Supplement: Supplementary file 4 — Mean scores for difficulties sleeping, symptoms and activities at daily points. (PDF 165 kb) [file 12955_2019_1138_MOESM4_ESM.pdf]

Estimating loss in quality of life associated with asthma-related crisis events (ESQUARE): a cohort, observational study
Additional file 6

*Figure 1: Mean scores for difficulties sleeping, symptoms and activities at daily time points*

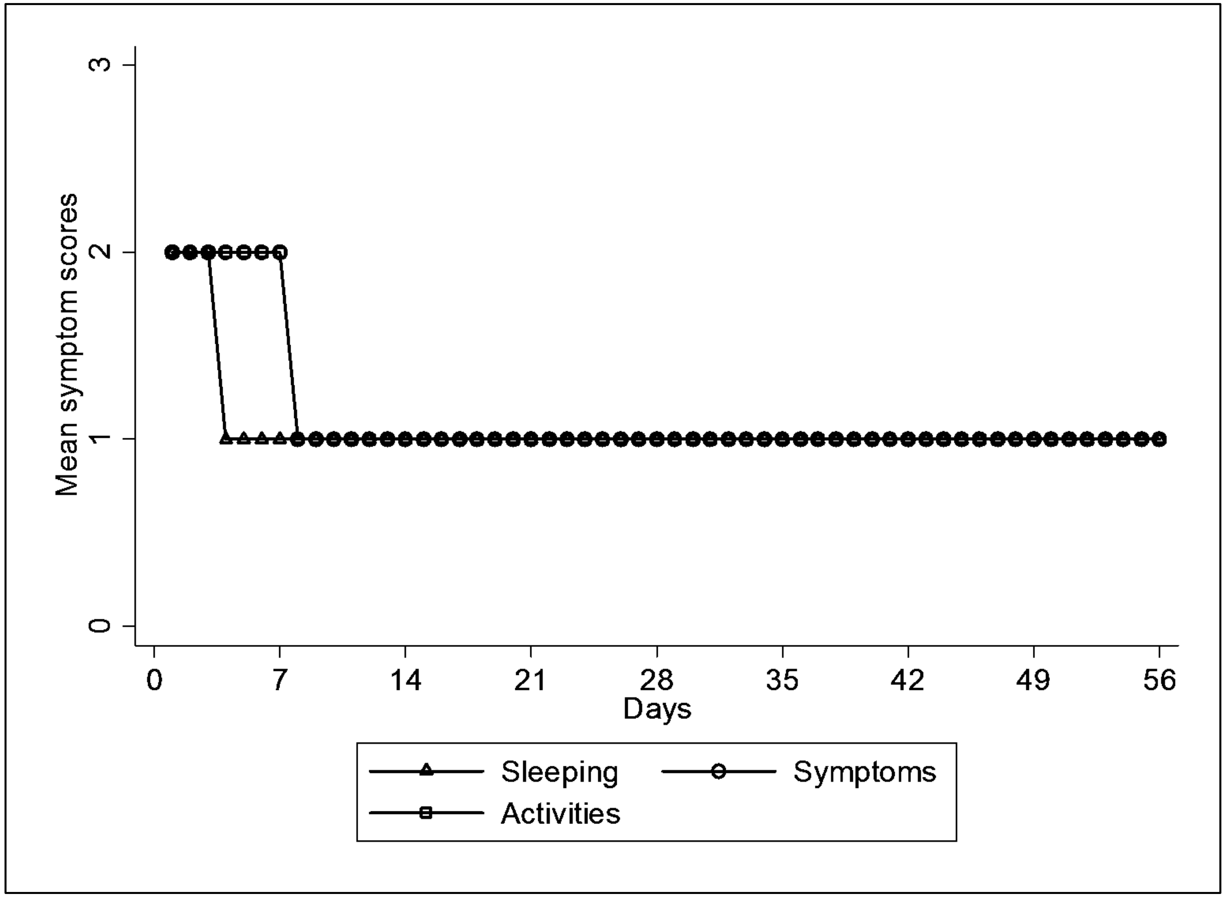


*y-axis: 0 = no symptoms, 1 = mild symptoms, 2 = moderate symptoms, 3 = severe symptoms*
