# Supplementary material for: Estimating loss in quality of life associated with asthma-related crisis events (ESQUARE): a cohort, observational study
Source: Health Qual Life Outcomes. 2019 Apr 11;17:58. doi: 10.1186/s12955-019-1138-5 (PMC6458613; doi:10.1186/s12955-019-1138-5)
Supplement: Supplementary file 5 — Week 4 statistics for each quality of life questionnaire. (PDF 151 kb) [file 12955_2019_1138_MOESM5_ESM.pdf]

Estimating loss in quality of life associated with asthma-related crisis events (ESQUARE): a cohort, observational study
Additional file 3

Table 1: Week 4 statistics for each quality of life questionnaire

Item	N	Mean	SD	Range	Response rates	Floor effects	Ceiling effects
**EQ-5D-5L (utility)**	71	0.740	0.264	-0.005 to 1.00	58.7%	0.00%	15.5%
**EQ-5D VAS score**	73	65.9	21.42	11.00 to 100.00	60.3%	0.00%	0.00%
**AQLQ overall score**	70	4.09	1.48	1.47 to 6.94	57.9%	0.00%	0.00%
**AQLQ Symptoms score**	85	3.34	2.12	0.00 to 7.00	70.2%	0.00%	0.00%
**AQLQ Activity score**	85	3.32	2.00	0.00 to 7.00	70.2%	0.00%	0.00%
**AQLQ Emotional score**	85	3.36	2.27	0.00 to 7.00	70.2%	0.00%	2.40%
**AQLQ Environmental score**	85	3.63	2.34	0.00 to 7.00	70.2%	0.00%	1.20%
**AQL-5D (utility)**	70	0.687	0.173	0.450 to 1.00	57.9%	0.00%	2.90%
**TTO (utility)**	87	0.820	0.264	0.000 to 1.00	77.7%*	0.00%	51.7%

*The response rate is based on the denominator being 112 due to only the participants based at the Norwich hospital site (NNUH) being asked the TTO questions. All of the other response rates for the PROMS were based on the denominator being 121 as this was the total number recruited across all hospital sites where each participant was asked to complete PROM questionnaires.
Ranges for PROMs: EQ-5D-5L (-0.281 to 1); EQ-5D VAS (0 to 100); AQLQ (0 to 7); AQL-5D (0 to 1); TTO (0 to 1).
